# MycoTWIN Working Group Discussion: A Multi-Actor Perspective on Future Research Directions for Mycotoxins and Toxigenic Fungi Along the Food and Feed Chain

**DOI:** 10.3390/foods13223582

**Published:** 2024-11-09

**Authors:** Martina Loi, Antonio Moretti, Vincenzo Lippolis, Hayrettin Özer, Ceyda Pembeci Kodolbas, Elif Yener, İlknur Demirtaş, Pilar Vila-Donat, Lara Manyes, Veronica M. T. Lattanzio

**Affiliations:** 1 Institute of Sciences of Food Production, National Research Council of Italy, Via Amendola 122/O, 70126 Bari, Italy; martina.loi@ispa.cnr.it (M.L.); antonio.moretti@ispa.cnr.it (A.M.); vincenzo.lippolis@ispa.cnr.it (V.L.); 2 TUBITAK MAM Food Institute Gıda Enstitüsü, 41470 Gebze, Turkey; hayrettin.ozer@tubitak.gov.tr (H.Ö.); ceyda.pembeci@tubitak.gov.tr (C.P.K.); elif.yener@tubitak.gov.tr (E.Y.); ilknur.demirtas@tubitak.gov.tr (İ.D.); 3 Laboratory of Food Chemistry and Toxicology, Faculty of Pharmacy, University of Valencia, 46010 València, Spain; pilar.vila@uv.es (P.V.-D.); lara.manyes@uv.es (L.M.)

**Keywords:** mycotoxin, food security, food safety, mycotoxigenic fungi, future research directions, mycotoxin management

## Abstract

Mycotoxin research is facing unprecedented challenges, starting from the urgent need to cope with the consequences of climate change, the global shortage of grain due to unstable political scenarios, and the major transformation of the supply chains after the COVID-19 pandemic. In this scenario, the mycotoxin contamination of human and animal foods is still unavoidable, thus representing a major challenge to global food security. Next to this, the shift to sustainable and circular food production might be accompanied by an increase in food safety issues involving mycotoxins, e.g., when new technologies are applied to reuse side streams from the food industry, it is not known if and how mycotoxins accumulate in these by-products. MycoTWIN is an EU-funded Horizon 2020 project which fosters knowledge transfer and scientific cooperation within the Mediterranean area, involving worldwide experts, decision makers, and stakeholders in the field of mycotoxigenic fungi and mycotoxins. The MycoTWIN project hosted working group meetings, whose aim was to propose operational plans and/or scientific strategic plans to shape the future research directions to better cope with these challenges. In the working group cycle “Future proof approaches for the management of toxigenic fungi and associated mycotoxins along the food chain”, a multi-actor group was guided in co-creation exercises to elaborate on future research directions and propose relevant actions to be implemented for the present to long-term time periods. The discussion focused on three main topics relevant to the assessment and management of risks associated with mycotoxins and toxigenic fungi: (i) needs for the harmonization of molecular and chemical methods and data analysis, (ii) from lab research to marketable solutions: how to fill the gap, and (iii) gaps in data quality for risk assessment.

## 1. Introduction

In recent years, the food system has been facing concurrent, emergent, and unexpected challenges, such as climate change, geopolitical tensions, the global shortage of grains, societal changes, and a major transformation of the supply chains after the COVID-19 pandemic. The capability of producing enough, safe, and nutritious food and feed for the growing global population is seriously affected, especially in countries already facing food safety and security issues.

A recent survey covering the first half of 2024 detected high mycotoxin prevalence and associated risks across North and Latin America, Europe, Sub-Saharan Africa, and Asia, while moderate risks were noted in Northern Europe and Oceania [1]. Besides regulated mycotoxins, emerging ones (such as fusaric acid, enniatins, culmorin, apicidin, butenolide, fusaproliferin, *Alternaria* toxins, auofusarin, emodin, beauvericin (BEA), diacetoxyscirpenol (DAS), moniliformin (MON), and sterigmatocystin) are increasingly detected worldwide in staple cereal commodities. The toxicity of these compounds, as well as their potential to interact with each other and with other xenobiotics, poses an even greater risk to human and animal health [2].

Indeed, despite the efforts of the scientific community and stakeholders, mycotoxins still represent the third most notified hazard category in Europe by the Rapid Alarm Safety System for Food and Feed (RASFF), with f.i. a 10.5% increase in 2022 compared to 2021 [3].

Constantly active research is in progress to develop advanced multi-mycotoxin detection methods for complex samples, as well as prevention strategies (including predictive modeling) and mitigation strategies through physical and biological approaches; however, due to the evolving scenario, several challenges persist. Building capacity for mycotoxin management, especially in resource-limited regions, and harmonizing efforts for effective risk assessment and mitigation is crucial. Achieving transitions towards a healthier, safer, sustainable, and environmentally friendly food system as set out in the Farm2Fork EU strategy [4], and counteracting the global threat of mycotoxin contamination, requires a bold and holistic response by the research community. One solid approach to creating future European Food Safety strategies is to identify the priorities of common interest and share the results, information, and knowledge for institutions and policy makers through cooperation initiatives.

Horizon 2020 was designed to promote scientific excellence, foster innovation, and address major societal challenges. The program prioritized fields such as climate action, sustainability, health, and digitalization, with a strong emphasis on involving business operators, fostering partnerships, and encouraging international cooperation to solve complex global issues. As part of the H2020 scope, during the EU H2020-funded project MycoTWIN [5], working group meetings were organized with the aim of proposing operational plan and/or scientific strategic plans to shape the future research directions in the field of mycotoxigenic fungi and mycotoxins. During the working group cycle “Future proof approaches for the management of toxigenic fungi and associated mycotoxins along the food chain” organized by CNR-ISPA, three trending topics were identified and discussed by the MycoTWIN working with a multidisciplinary, holistic, and collaborative approach provided by the hosting EU Food Safety Platform [6] (see Section 2 for full details).

When selecting the topics of the working groups, the choice was driven by an extensive review of the existing literature, engaging with scientific networks/research consortia (H2020-funded FoodSafety4EU and EFSA-funded MYCOBOOST projects), monitoring white papers and reports of government agencies (FAO and EFSA), and by the strategic insights gained from the round table discussions held during the first MycoKey International conference, titled “Global Mycotoxin Reduction in the Food and Feed Chain” [6,7,8]. The topics’ selection was conducted to ensure access to up-to-date information on the challenges, research priorities, pressing industry and regulatory issues, and emerging trends shaping the field of mycotoxin research.

Future research needs in the food safety domain are elaborated in recently issued research agendas setting the updated research and innovation framework (f.i. the Food 2030 agenda, and its pathway action “Food Safety Systems of the Future”) [9], or the FoodSafety4EU Strategic Research and Innovation Agenda [10]. In this framework, the generation of and access to Findable, Accessible, Interoperable, and Reusable (FAIR) data are acknowledged as the fundamentals for improving food safety management and risk assessment. Thinking about the rapid advances in toxicology and in the assessment of chemical safety in humans (e.g., mycotoxin mixtures) as well as the spreading of big data (large amount, complex, structured and unstructured data) in this field, the availability of improved and harmonized approaches to generate, analyze, interpret, reuse, and integrate data about mycotoxin contamination at national/EU level is of utmost importance.

The same consideration applies to the design of predictive and modeling tools needed, for instance, to cope with climate change’s impact on mycotoxin/toxigenic fungi distribution patterns.

Next to risk assessment, extensive and systematic mycotoxin/fungi monitoring either in the field or along the chain can contribute to the development and implementation of mitigation measures supporting risk management decisions. In this respect, space and funding for the translation of laboratory research into practical solutions, enabling food business operators to control and share data about contamination, can be found in the Horizon Europe program [11]. The established and emerging technologies for real-time mycotoxin/fungi monitoring, complemented by validated approaches to generate reliable data, are expected to support preventive measures and the reduction in food waste due to product non-compliance.

Based on all the above considerations, the following topics were selected to be elaborated by the working group: (i) Needs for harmonization of molecular and chemical methods and data analysis, (ii) From lab research to marketable solutions: how to fill the gap, and (iii) Gaps in data quality for risk assessment.

## 2. Methodology for Collaborative Exercise

### 2.1. Participants

Working group participants were selected among the MycoTWIN consortium, international experts, and the private sector according to diverse research expertise and capabilities, geographic origin, affiliation, and interests in the food safety domain. They represented: research institutions (National Research Council of Italy—Institute of Sciences of Food Production—CNR-ISPA—Bari, Italy; TUBITAK MRC Life Sciences—Gebze/Kocaeli, Turkey; and International Institute of Tropical Agriculture, The Institut National De Recherche Pour L’agriculture, L’alimentation Et L’environnement—INRAE—Bordeaux, France), universities (Valencia University—Valencia, Spain; Piacenza University—Piacenza, Italy; Parma University—Parma, Italy; Liège University—Liège, Belgium; Ghent University—Ghent, Belgium; and Cranfield University—Cranfield, UK), global food authorities (the European Commission, Joint Research Centre—JRC), the EU Food Safety Platform, and national and international companies (Bonifiche Ferraresi, Eurofins Tecna, and Syngenta).

The collaborative exercise was organized as a series of 2 working groups as described in Section 2.2 and Section 2.3; 37 and 11 participants were involved in phase 1 and phase 2, respectively.

### 2.2. Working Group 1 (1st Phase)

In the preparatory work executed before the working group, based on an extensive review of the available strategic documents (see Section 1), three relevant topics in the mycotoxin field were selected by the authors, and a draft list of issues for the working group participants was prepared.

The first working group was held as a live event on 22 May 2022. For each proposed topic and its related draft list of issues, the discussion was structured as follows:

1. *Silent reflection*: Each participant considered the proposed draft list of issues and annotated amendments or additions of new issues to be included in the subsequent discussion.
2. *Plenary discussion*: The discussion was led by a facilitator, who moderated the discussion, and a reporter, who took notes of the amendments and/or new issues generated by the participants.
3. *Voting and ranking*: Once the participants agreed on the list of issues generated from the plenary discussion, they were asked to vote on them according to the priority of action, with a scale of 1 (low priority) to 10 (high priority). To calculate the weighted mean, the total scores for each issue were divided by the number of participants who ranked that issue. The first six issues, prioritized according to the weighted mean, were subjected to another round of voting according to uncertainty using the same scale (1 to 10).

### 2.3. Working Group 2 (2nd Phase)

The second working group discussion was held as a hybrid event among MycoTWIN experts and external guests on 15 April 2024. It was structured as follows:

1. *Topic discussion*: The issues prioritized in the previous working group were briefly discussed, also reviewing the priority and uncertainty scores.
2. *Target action proposition*: For each prioritized issue, the participants were asked to propose a target action or strategic plan by specifying the related hazard. The participants’ contributions were first collected on a digital board and then reviewed and rephrased until consensus was achieved.
3. *Interlinks*: After carrying out step 2 for each of the three topics, the participants were asked to discuss and indicate interlinks between the proposed actions and subtopics.

Common actions or actions with multiple impacts selected with the interlink exercise were discussed considering urgency and uncertainty and classified into the following:

(i) Present (one-year needs): Urgent issues;
(ii) Near future (three-year needs): Emerging issues;
(iii) Normal term (five-year needs): Mid-term issues;
(iv) Long term (eight-year needs): Far future issues.

This was performed with the aim to provide input for future research in the field of mycotoxins and toxigenic fungi.

*Clustering issues within specified timeframes*: Scores for the prioritized issues (Section 2.2, item 3), based on votes for both the priority of action and level of uncertainty, were normalized on a 0–5 scale, with the highest voted issue across all three topics serving as the benchmark (score 5). The issues were clustered within the four timeframes (present—one-year needs, near future—three-year needs, normal term—five year-needs, and Long term—eight-year needs) based on a qualitative evaluation that, however, took into account not only the normalized scores, but also the experts’ evaluations of issues, i.e., the urgency (priority) of the identified needs, the uncertainty of the available solutions/methodologies to cope with them, and the possible interlinks among the issues.

### 2.4. Statistical Analyses

The priority of action and uncertainty rank scores (item 3, Section 2.2) were subjected to normality testing distribution using the Shapiro–Wilk test. Following the assessment of non-normal data distribution, the non-parametric Friedman test was applied, along with the post hoc Wilcoxon signed-rank test, to assess statistically significant differences among the voted issues. The Bonferroni-adjusted *p*-value was applied for pairwise comparisons. All the statistical analyses were performed using the IBM SPSS software (IBM SPSS Statistics for Windows, Version 29.0 (Armonk, NY, USA: IBM Corp, 2022).

### 2.5. Glossary

For the purpose of the working group meetings, the following glossary was adopted:

(i) High level of priority of action: Something given or meriting attention before competing alternatives since it represents a pre-requisite, or a key requisite, to undertake the other issues in the list;
(ii) High level of uncertainty: not fully investigated and researched; not based on well-established knowledge; values are unknown; possible outcomes are incomplete and ambiguous; unstable; hard to predict; difficult to estimate; unobvious impact.

## 3. Results

In working group 1 (first phase), for each selected topic, the current constraints, enablers, or requirements (from now on defined as “issues”) were identified by the experts in co-creation exercises and ranked according to the priority of action and uncertainty, according to the methodology described in Section 2.2. Working group 1 resulted in a list of six prioritized issues for each of the three discussed topics. In working group 2 (second phase), the prioritized issues were further explored, identifying the main related hazards and proposing actions to cope with them. Finally, the issues and relevant proposed actions were clustered in four timeframes according to the priority of action, uncertainty, and interlinks identified between the issues.

### 3.1. Topic 1: Needs for Harmonization of Molecular and Chemical Methods and Data Analysis

Harmonized, quality data are essential to ensure the consistency, comparability, and reliability of results. They represent a mandatory requirement to perform large-scale monitoring, gain insight into mycotoxin and fungal contamination, support the establishment or revision of mycotoxin regulatory limits in food and feed, and manage recommendations and guidelines at various levels. Nonetheless, given the current increase in data generation driven by high-throughput and AI-based approaches, harmonizing molecular and chemical methods and data analysis has become a critical issue that requires immediate attention from the research community.

#### 3.1.1. Topic 1 Issue Definition and Prioritization

During the first working group meeting, eleven issues related to constraints and enablers for method harmonization were identified (Table 1). The experts identified as major issues the need for standardization protocols/procedures (issues n. 1, 2, 7, 8, 9, and 10), need for reference material (issue n. 3), data generation and sharing (issues n. 4, 6, and 7), and method validation (issues n. 8 and 11). The ranked issues are shown in Table 1 (priority of action) and Table 2 (top six, uncertainty). Looking at the uncertainty ranking, the group’s view revealed that there are still gaps in traditional issues that need to evolve in the new scenario. These include reference material—with emphasis on multi-mycotoxin or emerging/modified mycotoxin reference materials—sampling and the validation of new analytical approaches.

#### 3.1.2. Definition of Hazards and Relevant Actions for Topic 1

The development of collaborative, data-driven platforms, infrastructures, and online repositories were identified as crucial actions to undertake. These platforms enable data sharing and use across different research fields and link molecular/predictive data to phenotype and bioanalysis, as also highlighted during the plenary discussion. In addition, simplified and harmonized data formats and management, also supported by artificial intelligence, were classified as emerging issues (Table 3).

Interestingly, while it is clear that new hazards have been identified for emerging mycotoxins and mycotoxins in biofluids and new commodities, such as fermented foods, novel foods, and side streams, the situation for toxigenic fungi is less clear as they are not regulated. A notable normal-term issue that emerged from the discussion regarded fungal taxonomy, for which there is the need for reference strains to define fungal chemotypes based on the mycotoxigenic potential rather than core features. Hence, fungal taxonomy should be revised according to the toxigenic potential, especially in light of climate change and the emergence of new commodities of interest.

### 3.2. Topic 2: From Lab Research to Marketable Solutions: How to Fill the Gap

Filling the gap between laboratory research and marketable solutions is a crucial task to ensure that scientific advancements are translated into tangible benefits for stakeholders and society. This process involves a multi-actor, multidisciplinary intervention to address the multifaceted challenges, different backgrounds, and perspectives. Indeed, often the information or efforts required to bring innovation into the market rely also on other stakeholders, such as large-scale and/or smallholder farmers, and observations made by extension agents, policy makers, and private industry, rather than on solely scientists [12].

#### 3.2.1. Topic 2 Issue Definition and Prioritization

Twelve issues related to constraints and enablers for bridging the gap between laboratory research and marketable solutions were identified (Table 4). The experts highlighted not only the need for improvements in technologies (issues n. 1, 3, 4, 6, 8, 9, 10, and 11), but also the need to bring awareness and a structured, improved communication (issue n. 2), and to engage with the regulatory bodies (issues n. 5 and 7) and companies (issues n. 2, 9, and 12). These latter were also listed among the most uncertain issues to tackle (Table 5), also due to the lack of well-established and trusted channels of communication between these actors of the food safety system, especially when thinking about local authorities and small enterprises/farmers. However, issues like the verification of the analytical process and full traceability were put on the basis of acceptance by regulatory bodies and trustful communication.

#### 3.2.2. Definition of Hazards and Actions for Topic 2

Among the actions to be undertaken in the present–near future, the experts highlighted the need for fitness-for-purpose approaches in method validation, performance, and verification protocols. They also emphasized the importance of promoting automated procedures and providing on-site training to improve reliability, validity, and quality control. Investments and cooperation were considered urgent actions as well.

As for normal-term improvements (over the next 5 years), the experts only identified technical improvements, also based on AI, to improve the ease of use and method reliability.

Concerning hazard identification, regulated mycotoxins and mycotoxin adducts/metabolites in the blood and urine emerged as important hazards, for which the gap from lab research to marketable solutions is still high.

### 3.3. Topic 3: Gaps in Data Quality for Risk Assessment

Continuous risk assessment is crucial to comprehensively understand and mitigate the potential adverse effects of mycotoxin contamination, thereby ensuring safe food and feed. High-quality data represent the foundation for each step of risk assessment, i.e., hazard identification and characterization, exposure assessment, and risk characterization.

Nonetheless, researchers consistently point out the limited availability, inconsistency, and lack of harmonization and integration in data concerning fungal and mycotoxin contamination and the need for robust methodologies and cutting-edge technologies for data collection, analysis, and interpretation [13].

#### 3.3.1. Topic 3 Issue Definition and Prioritization

Nine issues were identified by the experts (Table 6) regarding protocols and guidelines for data and metadata collection and sharing (issues n. 1, 2, 3, 6, and 7), platforms for data storage and management (issues n. 4, 5, and 9), and privacy/reputation (issue 8). The most urgent issues identified regarded harmonizing data and promoting data sharing (Table 7).

#### 3.3.2. Definition of Hazards and Actions for Topic 3

The actions identified by the experts in the near- and normal-term timeframe aimed at supporting data collection and curation, and platforms in which they should be transparently shared. These actions targeted users (involvement and training) and digital infrastructures (user-friendly and accessible software, workflows, and open access tools).

For the first time, long-term actions (8-year needs) were identified and focused on metadata. In the era of omics techniques, metadata are becoming as important as the data themselves, as they include the full description of how the data were obtained, processed, and annotated [14]. Besides actions aimed to establish harmonized protocols and formats, the experts highlighted the need to implement knowledge and awareness on metadata, their acquisition through New Approach Methodologies (in silico, in vitro, omics, predictive, and biomarker-based technologies), and their sharing among researchers, risk assessors, and risk managers.

## 4. Discussion

In relation to the three discussed topics, a number of strategic actions was proposed to address relevant hazards that included regulated and emerging mycotoxins as well as mycotoxigenic fungi (Table 3, Table 8 and Table 9).

**Figure 1:**
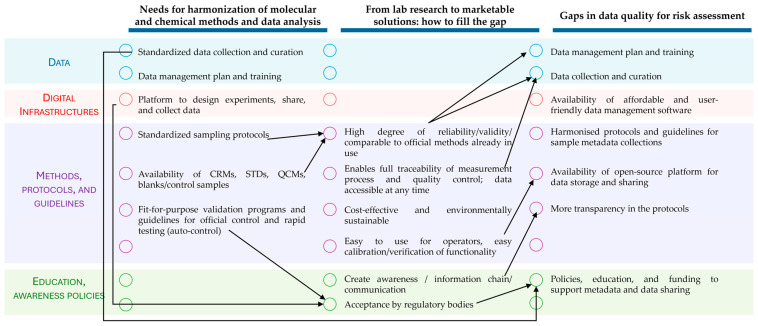
Simplified scheme of the topic interlinks proposed by the MycoTWIN working group. For each topic (column), the issues were categorized by color: Blue—issues related to data; Orange—issues related to digital infrastructures; Purple—issues related to methods, protocols, and guidelines; Green—issues related to education, awareness, and policies. The arrows indicate the interlinks among the issues of different topics.

The issues emerging from Topic 1 were among the ones with a higher impact on the issues of the other topics. Indeed, method harmonization is often a prerequisite to implementing the transferability of research to the market (Topic 2) and to defining data used in risk assessment (topic 3). A consistent methodology, which includes standardized data collection and curation, the availability of reference material, and fit-for-purpose validation programs, enables one to easily use and implement novel methods into the market and verify them against the official ones (Figure 1). Sharing data was also regarded as a tool to create awareness and promote communication among research, private companies, and consumers, which in turn fosters transparency, collaboration, and trust-building (Figure 1). Regulatory bodies support the strategic goals of the European Union, encouraging innovation and scientific advancement. Their role in promoting policies, education, and funding to support metadata and data sharing was acknowledged (Figure 1). The statistical analyses revealed significant differences only among the priority scores of Topics 1 and 2, while no differences were found in their uncertainty scores or among all the Topic 3 scores. Overall, the closely grouped scores—including those in which statistically significant differences were observed—indicated that the working group participants assigned a consistent level of recognition to the issues for all the topics. Their role in promoting policies, education, and funding to support metadata and data sharing was acknowledged (Figure 1). It is important to note that the scores were assigned individually, reflecting the individual participants’ experience and expertise. To reach a consensus, the issues were further elaborated within the working group taking into account the scoring results. By elaborating on (i) the urgency (priority) of the identified needs, (ii) the uncertainty of the available solutions/methodologies to cope with them, and (iii) the interlinks showing which actions were pre-requisite for others, the experts clustered the proposed actions in four timeframes (present, near, medium, and long term) to envisage a possible strategic action plan. The actions to be undertaken in the short–medium terms were identified mainly in Topics 1 and 2. The discussion highlighted that advanced research on regulated mycotoxins (and relevant fungi) and emerging ones is already in progress. The needed actions deal mainly with better exploitation/transfer and sharing of already available research results. Therefore, approaches and guidelines for the harmonization and standardization of data and protocols, which in turn will enable data sharing and joint exploitation, resulted to be of high priority.

A major impact will result from the increasing application of AI-based approaches in mycotoxin risk prediction, characterization, assessment, and management. In this respect, full compliance with the FAIR principles in data generation, collection, and storage will require actions in the medium–long term to cope with big data and issues related to Intellectual Property (IP) and data curation.

Common issues in Topics 1 and 3 highlighted the need for improving data collection, curation, and platforms/software for their management. Initiatives led by the World Health Organization (WHO) jointly with the Food and Agriculture Organization (FAO), and EFSA have made several progresses towards the definition of descriptors for mycotoxin contamination data [15]. Nonetheless, the occurrence of mycotoxigenic fungi in food and feed remains largely unregulated. Although there is a significant body of literature addressing the occurrence of these fungi in specific commodities and in specific geographical areas, the data reported in these studies are not systematically reported nor analyzed, often resulting in fragmented or inconsistent data. In addition, the current taxonomic definition for fungal species is not adequate to describe the toxigenic potential of a fungal strain in a specified matrix in a geographical area under a defined climatic condition. Harmonized approaches to identify mycotoxigenic fungi would facilitate more effective monitoring, risk assessment, and management of fungal contamination along the supply chain.

A cross-cutting need over the three topics resulted to be the availability of platforms and infrastructures for data storage, knowledge sharing, and access. This addresses not only mycotoxins (food safety and risk assessment) but also fungi, since further developments were advocated to establish and maintain international microbial collections as well as new and harmonized approaches for species classifications or re-definition based on their mycotoxigenic potential. Capitalizing and making the already existing platforms interoperable is an action that can be planned in the short term, while the achievement of fully open-source solutions is envisaged in the long term.

Long-term issues were identified within Topic 3 and deal with (i) the harmonization of data, metadata, and protocols for emerging risk assessment and toxicological assessment methodologies (NAMs) and (ii) long-term strategies to shape future policies and orienteering future funding schemes and promoting investments, also through education and training. The latter is expected to contribute to facilitating the relationship between risk assessors and risk managers with a view to the evolving food safety scenarios.

## 5. Conclusions

Starting from the available knowledge, a co-creation process was implemented, along with a workshop series, to generate a shared vision of the challenges related to the management of food safety risks, arising from mycotoxin and toxigenic fungi contamination along the food/feed chain. The multi-actor composition of the working groups allowed them to capture stakeholders’ needs, fostering a multi-perspective approach in prioritizing and undertaking these challenges.

In line with the main objective of the MycoTWIN project, which was capacity building, this collaborative exercise might facilitate the incorporation of new researchers into the group and foster collaborative networks around topics of common interest, while the outcomes of the discussion are expected to serve the scientific community by guiding authors in developing new research projects.

By identifying common actions or actions with multiple impacts on the prioritized issues, the MycoTWIN working group selected and proposed three emerging research topics in the field of mycotoxigenic fungi and mycotoxins for the European framework.

(1) Further development of in silico models for emerging risk assessment (NAMs), including harmonized models and protocols for computational modeling, high-throughput screening, omics technologies, and mechanistic toxicology;
(2) To develop new and more robust approaches for the taxonomic classification of fungal species, also based on interlaboratory comparisons;
(3) To map the available resources and propose pathways for the integration and interoperability of the existing platforms/infrastructures connecting data and metadata from different sources (i.e., genomic analyses, transcriptomic studies, biological assays, expression profiles, and more).

If complemented by adequate measures for awareness building and training, future research in these domains is expected to provide input for future food safety R&I programs.

## Figures and Tables

**Table 1:** Prioritized issues (priority of action) for Topic 1. Priority scores range from 1 (low priority) to 10 (high priority).

Needs for Harmonization of Molecular and Chemical Methods and Data Analysis *For Each Issue, What Is the Perceived Level of Priority of Action?*		
Issue n.	Issue	Priority Score ^1^
**1**	Standardized sampling protocols	6.00 a
**2**	Standardized data collection and curation	5.48 a
**3**	Availability of certified reference materials (CRMs), standards (STDs), quality control materials (QCMs), and blanks/control samples	5.41 ab
**4**	Data management plan and training	5.41 ab
**5**	Fit-for-purpose validation programs and guidelines for official control and rapid testing	5.03 ab
**6**	Platform to design experiments, share, and collect data	5.03 ab
**7**	Simplified standard data format	4.79 ab
**8**	Measurement uncertainty protocols	4.64 ab
**9**	Criteria for calculating and evaluating Limit Of Quantification (LOQ)	4.25 b
**10**	Guidelines and acceptance criteria for matrix effects	4.14 b
**11**	Availability of interlaboratory ring trials (IRTs) and/or proficiency testing programs (PTPs)	4.11 b

^1^ letters indicate statistically significant differences among issues (Bonferroni-adjusted *p*-value < 0.000758).

**Table 2:** Prioritized issues (priority of action and uncertainty) for Topic 1. Uncertainty scores from 1 (low uncertainty) to 10 (high uncertainty).

Needs for Harmonization of Molecular and Chemical Methods and Data Analysis. *For Each Proposition, What Is the Perceived Level of Uncertainty?*	
Issue	Uncertainty Score ^1^
Availability of certified reference materials (CRMs), standards (STDs), quality control materials (QCMs), and blanks/control samples	3.91
Standardized data collection and curation	3.69
Standardized sampling protocols	3.39
Fit-for-purpose validation programs and guidelines for official control and rapid testing (auto-control)	3.36
Data Management plan and Training	3.35
Platform to design experiments, share, and collect data	2.99

^1^ no statistically significant difference emerged among issues (Friedman’s *p*-value = 0.477).

**Table 3:** Hazards and relevant actions proposed by the MycoTWIN working group participants for each prioritized issue of Topic 1—needs for the harmonization of molecular and chemical methods and data analysis.

Time	Issue	Hazard	Action
Present (1-year needs)	Platform to design experiments, share, and collect data for feed models	All mycotoxins Re-occurring mycotoxins related to climate change (f.i. aflatoxins)	Starting from the already existing ones, to develop or integrate collaborative infrastructures/data-driven platforms/online repositories Include data for bioanalysis for evidence based on public health information
Near Future (3-year needs)	Standardized data collection and curation	Emerging mycotoxins Modified mycotoxins Regulated mycotoxins in new commodities of interest	To develop simplified standard data formats, taking into account big data and AI developments as well as the FAIR ^1^ principles To develop training modules on data collection and curation for different target users To develop committee-supervised, unanimous consensus on methods/approaches used to obtain data for taxonomic rearrangements
		Fungal chemotypes vs. fungal species	To enforce the definition of “chemotypes” rather than species (mycotoxin production vs. core genome) To promote investments in risk monitoring
	Standardized sampling protocols	Regulated mycotoxins in regulated and unregulated commodities of interest (f.i. fermented foods, novel foods, and side stream) Fungi	To develop and/or standardize sampling methods for new commodities To develop and/or standardize alternative sampling methods (f.i. based on the latest developments in dust sampling) To develop and/or feed infrastructures and culture collections, including methodologies to define and maintain reference strains
	Fit-for-purpose validation programs and guidelines for official control and rapid testing (auto-control)	Regulated mycotoxins in regulated commodities	To develop validation programs, including in-house validation protocols for rapid testing—co-development with food business operators To develop dedicated proficiency tests To propose an update of the Regulation 519/2014/EC (mycotoxin screening methods)—link with EFSA ^2^ to take into account risk assessment needs
		Fungi	For the classification of new species: create more links between biology/genetics and taxonomy (structured pathways)
	Data Management plan and Training	Regulated and (re)emerging mycotoxins	To propose harmonized data formats To implement AI ^3^/machine learning approaches to merge redundant data/integrate the existing data To develop joint training modules for AI ^3^ experts and analytical chemists
		Fungi	To develop AI/machine learning for modeling—investigate the link between genomic elements To develop AI-based approaches to predict the biology of a microorganism
Normal term (5-year needs)	Availability of CRMs ^4^, STDs ^5^, QCMs ^6^, and blanks/control samples	Emerging mycotoxins Modified mycotoxins Regulated mycotoxins in new commodities of interest (f.i. fermented foods, novel foods, and side streams)	To develop new protocols for CRM ^4^, STDs ^5^, and QCMs ^6^—go for the fitness-for-purpose approach To develop standard methods/procedures for reference material characterization Define reference values (low vs. high exposure) for mycotoxin bioanalysis
		Fungal chemotypes (defining mycotoxigenic potential)	Define reference strains for fungi Define robust taxonomy classification through interlaboratory comparison—long-term perspective

^1^ FAIR: Findable, Accessible, Interoperable, and Reusable; ^2^ EFSA: European Food Safety Authority; ^3^ AI: artificial intelligence; ^4^ certified reference materials (CRMs); ^5^ standards (STDs); ^6^ quality control materials (QCMs).

**Table 4:** Prioritized issues (priority of action) for Topic 2.

From Lab Research to Marketable Solutions: How to Fill the Gap *For Each Proposition, What Is the Perceived Level of Priority of Action?*		
	Subtopic/Issue/Proposition	Priority Score ^1^
**1**	Cost-effective	5.75 a
**2**	Create awareness/information chain/communication	5.67 a
**3**	Easy to use: limited expertise and minimal operator manipulations/easy calibration/verification of functionality	5.64 a
**4**	High degree of reliability/validity/comparable to official methods already in use/brand-independent transferability	5.42 ab
**5**	Acceptance by regulatory bodies	5.3 ab
**6**	Enables full traceability of measurement process and quality control; data accessible at any time	5.18 ab
**7**	Compliant with regulations/standards/contracts below regulation	5.18 ab
**8**	High degree of reliability/validity/comparable to official methods already in use	5.1 ab
**9**	Secure supply of materials	4.9 ab
**10**	“Green” technology, sustainable, safe, and easy handling	4.82 ab
**11**	Enables process automation and speed, online capability	4.78 ab
**12**	Productability studies	4.26 b

^1^ letters indicate statistically significant differences among issues (Bonferroni-adjusted *p*-value < 0.000758).

**Table 5:** Prioritized issues (priority of action and uncertainty) for Topic 2.

From Lab Research to Marketable Solutions: How to Fill the Gap *For Each Proposition, What Is the Perceived Level of Uncertainty?*	
Subtopic/Issue/Proposition	Uncertainty Score ^1^
Easy to use: limited expertise and minimal operator manipulations/easy calibration/verification of functionality	3.91
Enables full traceability of measurement process and quality control; data accessible at any time	3.66
Acceptance by regulatory bodies	3.55
Create awareness/information chain/communication	3.23
High degree of reliability/validity/comparable to official methods already in use	2.97
Cost-effectiveness	2.58

^1^ no statistically significant difference emerged among issues (Friedman’s *p*-value = 0.082).

**Table 6:** Prioritized issues (priority of action) for Topic 3.

Gaps in Data Quality for Risk Assessment *For Each Proposition, What Is The Perceived Level of Priority of Action?*		
	Issue	Priority Score ^1^
**1**	Harmonized protocols and guidelines for sample metadata collections	5.86
**2**	Policies, education, and funding to support metadata and data sharing	5.73
**3**	Data collection and curation	5.63
**4**	Availability of open-source platforms for data storage and sharing	5.53
**5**	Availability of affordable and user-friendly data management software	5.39
**6**	More transparency in the protocols	5.31
**7**	Simplified standard data format	5.04
**8**	Privacy/reputation	4.96
**9**	Increased knowledge of data management plans	4.86

^1^ no statistically significant difference emerged among issues (Friedman’s *p*-value = 0.091).

**Table 7:** Prioritized issues (priority of action and uncertainty) for Topic 3.

Gaps in Data Quality for Risk Assessment *For Each Proposition, What Is the Perceived Level of Uncertainty?*	
Prioritized Issue	Uncertainty Score ^1^
Harmonized protocols and guidelines for sample metadata collections	5.00
Policies, education, and funding to support metadata and data sharing	4.79
More transparency in the protocols	4.03
Availability of open-source platforms for data storage and sharing	3.74
Availability of affordable and user-friendly data management software	3.70
Data collection and curation	3.46

^1^ no statistically significant difference emerged among issues (Friedman’s *p*-value = 0.051).

**Table 8:** Hazards and relevant actions proposed by the MycoTWIN working group participants for each prioritized issue of Topic 2—from lab research to marketable solutions: how to fill the gap.

Time	Issue	Hazard	Action
**Present** **(1-year needs)**	High degree of reliability/validity/comparable to official methods already in use	Regulated mycotoxins or under consideration for future regulation (recommended)	To develop fitness-for-purpose method verification strategy assessment
	Cost-effectiveness and environmental sustainability	Regulated mycotoxins or under consideration for future regulation (recommended)	To promote investments in research and innovation/collaborative research projects with academia and industries To develop environmentally safe kits (no plastic—eco-friendly)
**Near Future** **(3-year needs)**	Enables full traceability of measurement process and quality control; data accessible at any time	Regulated mycotoxins	To develop fully automated procedures with remote access to the data and integrate AI-based technologies
	Acceptance by regulatory bodies	Regulated mycotoxins	To develop fit-for-purpose validation/performance verification protocols for operators (industries) To provide on-site training tailored to specific industry/operator needs To develop guidelines for dossier/documents to be submitted to the regulatory bodies
		Fungi	*Note:* fungi are not regulated, so no need for acceptance by regulatory bodies. However, the early detection of fungi should be in the regulation/recommendation coupled with accurate prediction approaches and mitigation steps
	Create awareness/information chain/communication		To develop and pilot innovative/simple communication models to reach operators/consumers in rural areas with low levels of education To create awareness of the importance of the early detection of fungi to promote mitigatory practices
**Normal term (5-year needs)**	Easy to use: limited expertise and minimal operator manipulations Easy calibration/verification of functionality	Regulated mycotoxins in regulated commodities and unconventional matrices (blood/urine) Mycotoxin adducts/metabolites in unconventional matrices (blood/urine), e.g., Ochratoxin A in blood, aflatoxins–lysin adducts	To develop green and simplified sample preparation protocols, including protocols without extraction (e.g., Infrared Spectroscopy) To improve test readers/detectors with user-friendly interface and online data management and to integrate user-friendly AI—simplified AI interfaces and more intuitive interfaces hiding the technical complexities of machine learning Implement method validation/verification protocols to assure accuracy, completeness, consistency, and reliability of obtained data

**Table 9:** Hazards and relevant actions proposed by the MycoTWIN working group participants for each prioritized issue of Topic 3—gaps in data availability and quality for risk assessment.

Time	Subtopic	Hazard	Action
**Near Future** **(3-year needs)**	Data collection and curation	Emerging mycotoxins Modified mycotoxins Regulated mycotoxins in new commodities of interest	To develop training modules on data collection and curation according to the FAIR ^1^ principles for different target users To develop and/or make accessible infrastructures for data collection, taking into account big data and AI ^2^ developments
**Normal term (5-year needs)**	More transparency in the protocols	All mycotoxins and fungi	Propose methodologies and strategies to reinforce trust and IP ^3^/ownership, like rights recognition and data anonymization
	Availability of open-source platforms for data storage and sharing	All mycotoxins and fungi	To map and propose pathways for the integration of the existing platforms to make data available and interoperable To develop approaches for data curation and data security and/or building awareness of the existing ones Propose clear definitions and roles in data responsibility and management
	Availability of affordable and user-friendly data management software	All mycotoxins and fungi	To develop user-friendly and cost-effective data management software To integrate in a user-friendly workflow open access tools for data management/processing
**Long term** **(8-year needs)**	Harmonized protocols and guidelines for sample metadata collections	Emerging mycotoxins Modified mycotoxins Regulated mycotoxins in new commodities of interest	To map the existing formats/protocols and guidelines and propose harmonized formats (f.i. commodity-dedicated) To develop harmonized protocols and guideline protocols for NAM ^4^ technologies (computational modeling, high-throughput screening, omics technologies, and mechanistic toxicology)
	Policies, education, and funding to support metadata and data sharing	All mycotoxins and fungi	To develop and implement training programs for risk assessors and risk managers about the latest scientific developments in relation to emerging risk identification, monitoring, and assessment To build awareness about in silico models for risk assessment (NAM ^4^) To enable the scientific community to share data/acknowledge data To establish revenues arising from data access and sharing To set up links with the existing microbial collections and to establish an international data/microbial collection-sharing platform through liasons/bilateral agreements between national authorities To promote investments in data-driven innovation

^1^ FAIR: Findable, Accessible, Interoperable, and Reusable; ^2^ AI: artificial intelligence; ^3^ IP: Intellectual Property; ^4^ NAM: New Approach Methodologies.

## Data Availability

The original contributions presented in the study are included in the article, further inquiries can be directed to the corresponding author.
